# Alzheimer's disease blood-based biomarker testing: A stakeholder-informed assessment of coverage considerations

**DOI:** 10.1177/13872877251329394

**Published:** 2025-04-01

**Authors:** Patricia A Deverka, Jalayne J Arias, Grace A Lin, Jessica Zwerling, Kathryn A Phillips

**Affiliations:** 1 Deverka Consulting, LLC, Durham, USA; 2 Center for Translational and Policy Research on Precision Medicine (TRANSPERS), Department of Clinical Pharmacy, University of California, San Francisco, San Francisco, CA, USA; 3 Department of Public Health and Behavioral Sciences, School of Public Health, Georgia State University, Atlanta, GA, USA; 4 Institute for Health Policy Studies, University of California, San Francisco, San Francisco, CA, USA; 5 Department of Neurology, Albert Einstein College of Medicine/Montefiore Medical Center, Bronx, NY, USA

**Keywords:** Alzheimer's disease, clinical utility, diagnostics, health equity, payer coverage

## Abstract

**Background:**

Recently published clinical studies suggest that blood-based biomarker tests (BBMTs) for Alzheimer's disease (AD) provide value, but in the U.S., neither public nor private payers currently cover these tests.

**Objective:**

To describe considerations for payer coverage of AD BBMTs that would need to be addressed to facilitate timely diagnosis and equitable patient access if clinical utility is demonstrated.

**Methods:**

We performed a targeted literature review to characterize predictable coverage barriers for BBMTs and inform the development of an interview guide. We conducted semi-structured interviews with clinicians, researchers, test developers, and a patient advocate and former payer (N = 12) to assess the barriers and refine the proposed key considerations for obtaining payer coverage.

**Results:**

Stakeholders noted that payers will require evidence of clinical validity and utility of BBMTs as part of their coverage determinations contingent on the specific indication for testing, with insufficient evidence for screening applications currently. Stakeholders also agreed that there are evidence gaps for use of BBMTs in patients from ethnic and racial minority communities that must be addressed. Given the shortage of memory specialists, stakeholders noted that limiting testing coverage authorization to specialists could be harmful to patients, particularly the underserved. Interviewees also agreed that patients with mild cognitive impairment or early-stage AD could benefit from earlier diagnosis to avoid progressing to moderate disease and limiting eligibility for new disease-modifying therapies.

**Conclusions:**

If BBMTs meet criteria for clinical utility, anticipating and planning for coverage and reimbursement before widespread implementation will be critical to ensuring broad, equitable access to BBMTs.

## Introduction

Current biomarker-based guidelines for Alzheimer's disease (AD) diagnosis rely on neuroimaging to detect amyloid plaques (amyloid positron emission tomography [PET]) and cerebrospinal fluid (CSF) biomarker testing.^1^ However, many patients may lack access to these tests, particularly in the primary care setting, where the diagnosis of AD overwhelmingly occurs.^2^ Therefore, a large proportion of the approximately seven million Americans over the age of 65 with AD are either not formally diagnosed or misdiagnosed.^3^ This is a particular problem among racial and ethnic minorities—non-Hispanic Blacks and Hispanics are more likely to experience a missed or delayed dementia diagnosis compared to Whites, resulting in more advanced disease when diagnosed.^4^ Timely, early, and accurate diagnosis of AD is becoming even more critical for patient management with the development and approval of new disease-modifying therapies (DMTs) such as monoclonal antibodies (e.g., lecanemab and donanemab).^5^

The development of blood-based biomarker tests (BBMTs) has created the opportunity to potentially facilitate diagnosis of AD at earlier stages, since blood tests are likely to be more widely accessible and easier to scale as compared to cerebrospinal fluid (CSF) testing or amyloid PET scans. Recent studies confirm potential advantages of BBMTs compared to both clinical and biomarker-based diagnostic tools.^6,7^

At least five BBMTs are on the market in the U.S.; however, none are currently recommended in clinical practice guidelines, nor do private or public payers cover them. Public and private payers typically base coverage decisions for new tests on an evaluation of the validity of the test (accuracy with which a test can reliably measure the analyte of interest and predict the presence or absence of a clinical condition) and evidence of clinical utility (the effects of testing on clinical management decisions or net patient health outcomes). Historically, innovative tests have made it to market despite the lack of evidence of clinical utility, leading to variation in and/or restrictive coverage policies.^8,9^ For example, the coverage environment is currently unclear for both CSF tests and amyloid PET scans, in part due to the fact that the tests were marketed and used prior to establishment of clinical utility. Currently, coverage of CSF AD biomarker testing is often implicit (e.g., bundling payment for the test with lumbar puncture procedure codes) or even explicitly not covered except in certain situations (e.g., the patient is being evaluated for a disease-modifying therapy and cannot access an amyloid PET scan). The recent change by Medicare leaving coverage decisions for amyloid PET scans to local Medicare contractors has also resulted in confusion amongst clinicians and patients regarding the financial implications of pursuing these tests as part of routine care.^10^

There is an acute need to identify key coverage issues so that payers can avoid replicating the current coverage pathway confusion when considering coverage for BBMTs. In the U.S, these tests are already marketed and being ordered today as laboratory developed tests (tests that are designed, manufactured and used in a single lab) without first gaining FDA approval. In addition, even when a new test may be covered by payers, utilization management approaches such as prior authorization or restricting test ordering to specialists may be considered and employed, as is the case for blood-based circulating tumor DNA for cancer care.^11^ We are not proposing that such tests be covered if they have not been shown to have clinical utility, but prior experiences with other technologies have demonstrated that coverage issues should be considered early to facilitate informed evidence development and timely coverage decisions.^12,13^

The purpose of this study is to assess stakeholder considerations for payer coverage of AD BBMTs. We first characterized predictable coverage barriers based on a targeted literature review and developed potential approaches for addressing these barriers. We then conducted semi-structured interviews with key stakeholders to validate the barriers and refine the proposed key considerations for obtaining payer coverage. We focused on clinicians, since they will be the frontline of ordering BBMTs. Our research questions were: (1) what evidence gaps need to be addressed in the near term?; (2) how might payer BBMT coverage policies mitigate or exacerbate equitable access to AD diagnosis and clinical management, including new drug treatments?; and (3) what are the most important and feasible payer considerations?

## Methods

### Targeted literature review

We undertook a targeted literature review to describe likely payer evidence hurdles and inform the development of a semi-structured interview guide. For purposes of this study, BBMTs include amyloid-β proteinopathies (e.g., Aβ_42_, Aβ_40_) and phosphorylated tau proteins measured individually or as part of panel testing that may also include biomarker ratios.^14,15^ Searches for this narrative review focused primarily on systematic reviews of BBMTs published in the past five years, as well as the evidence cited in the most recent Alzheimer's Association (AA) guidelines based on the ATN (amyloid-β [Aβ], tau, neurodegeneration) framework for diagnosing and staging AD based on core biomarker testing. We also included consensus statements by multi-stakeholder professional groups for use of BBMTs in clinical practice. (See Supplemental Material)

Publications were assessed for key evidence gaps and clinical practice challenges, particularly in underserved populations. Findings from this review and study team knowledge of the evidence requirements and processes for payer coverage policy informed development of a semi-structured interview guide to be used in the next phase of the study.

### Interview guide development

A draft version of the guide was piloted and tested with a neurologist for accuracy and comprehensiveness. The final version of the guide (see Supplemental Material) covered topics such as insurer coverage challenges for BBMTs compared to PET scans and CSF tests and whether and how payer BBMT coverage policies might affect equity and patient access considerations. We utilized primarily open-ended questions; however, interviewees were also asked to rate the importance and feasibility of payer considerations developed by the study team based on past experience with payer decision-making.

The interview guide focused on use cases for BBMTs in the diagnosis and management of AD. Evaluation of the clinical validity and clinical utility of a new test depends on the indication for testing. We described three specific use cases for the purpose of assessing payer coverage considerations. First, use of BBMTs for making the diagnosis of AD in symptomatic patients 65 years or older without requiring follow-on testing using amyloid PET or CSF testing. This use case assumes that BBM testing occurs in the context of a comprehensive clinical assessment of the patient's medical conditions and examination for other causes of cognitive impairment. A more specific version of this use case is to qualify patients for treatment with a DMT such as lecanemab. A second use case is as a “triage” test in symptomatic patients to determine whether confirmatory testing by amyloid PET or CSF is indicated. The third use case is as a screening test in asymptomatic older patients (e.g., above the age of 55 years, the lower age limit for some BBMTs in symptomatic patients).

### Semi-structured interviews

We recruited a convenience sample of 12 stakeholders based on their expertise, publications, and desired perspectives - neurology, geriatrics, internal medicine, patient advocacy, health equity, BBMT developer, former payer (Medicare)/pathologist. A total of 16 stakeholders were contacted and 12 stakeholders (see Supplemental Material), agreed to participate in the interviews, which were recorded for accuracy of note-taking purposes. One author (PD) with expertise in both qualitative research methods and precision medicine diagnostics conducted interviews between January–March 2024. Interviews were transcribed and the content coded deductively based on the interview guide.

Stakeholders were asked to provide their perspectives regarding the evidence gaps that need to be addressed in the near term and how payer BBMT coverage policies could mitigate or exacerbate equitable access to AD diagnosis and clinical management, including access to new drug treatments (at the time of the study, only lecanemab was practicably available as a disease-modifying therapy since as of January 2024 development of aducanumab was no longer supported by the company and therefore functionally not available for clinical use). Stakeholders were also asked to weigh in on the most important and feasible payer considerations from a clinical perspective. Given that BBMTs are an emerging technology, we also asked stakeholders to reflect on the likely impact on health equity.

Thematic analysis of interview notes was performed, organized by the specific topics and related codes covered in the interview guide. Results from the analysis of interview transcripts were discussed by all members of the study team. We then applied both public and private payers’ coverage frameworks based on our team's experience in analyzing payer coverage policies and payer decision-making for other precision medicine tests.^16^ During these discussions all summary qualitative data had the names and other identifiers removed. The results reported reflect the majority opinions of our experts, except as noted.

The UCSF IRB determined that the study was exempt.

## Results

Our literature review yielded a number of distinctive opportunities and challenges that BBMTs present for clinical practice.^17–34^ For example, because of their ease of use, acceptability to patients, and likely greater accessibility, BBMTs have the potential to be used more often for AD diagnosis than either CSF testing or amyloid PET scans. However, not all BBMTs have acceptable test performance characteristics compared to CSF tests and there are more evidence gaps regarding test interpretation for minority populations compared to whites. Also, given the current and predicted future shortage of specialists such as neurologists, primary care physicians (PCPs) will be the likely users of BBMTs if the tests are to be scaled in the current U.S. healthcare delivery system. These features led to our hypothesized clinical and payer implications (Table 1) and informed our list of coverage considerations that we discussed during interviews with stakeholders (Table 2).

**Table 1.** AD blood-based biomarker test (BBMT) features and relevant clinical and payer implications.

**BBMT Test Features**	**Potential Clinical Implications^a^**	**Potential Payer Implications^b^**
Variable test performance characteristics depending on particular assay	Benefits/harms differ by particular test panel, particularly with respect to diverse patient populations Minimal acceptable performance standards have been published by multi-stakeholder group for specific use cases and practice setting. Labs with high quality tests need to publish validation data compared to CSF testing in diverse patient populations	Will expect evidence of clinical validity and utility May use PA to limit testing to specific clinical contexts May only cover specific tests, e.g., tests with FDA approval May wait for practice guideline groups to independently recommend BBMT testing before covering Medicare may refer evaluation to MolDx as proteomic tests are part of established expertise
Less invasive and more accessible than alternative testing modalities	Simple blood draw facilitates ruling out AD as etiology of cognitive impairment assuming high NPV of BBMTs Assuming willingness to be formally diagnosed, patients are more willing to undergo a blood test than more invasive tests Patients in underserved areas will have fewer barriers to AD diagnosis	Payers may be concerned about reclassifying “normal aging” diagnosis to MCI due to AD/early AD May also be concerned about increasing numbers of patients eligible for DMTs Will need education re use of biomarker testing to diagnose AD
May be used as a CDx for DMTs such as Beta-amyloid monoclonal antibody therapies (e.g., lecanemab)	Neurologists, geriatricians and other specialists will treat patients with DMTs, not PCPs Specialists already familiar with using imaging and LPs to confirm diagnosis of AD if required	Documentation of amyloid pathology a requirement for obtaining access to DMTs, although test modality not specified Typically do cover PET scans or CSF testing when used to determine DMT eligibility Explicit coverage decisions re BBMTs would facilitate patient access to accurate AD diagnosis and DMTs
BBMTs only biomarker modality that can be readily used by PCPs and scaled rapidly	Would address limited access to AD biomarker testing in primary care PCPs very familiar with adopting new tests, but will require education re specialty referrals and follow-up requirements for indeterminate results BBMT tests likely to have slower adoption in underserved populations given lack of data in diverse populations regarding test performance and acceptability by patients	Benefits of BBMTs greatest in early-stage AD patients, which tend to be younger and may be covered by private payers Limiting use of BBMTs to specialists will be detrimental to patient access to an early, accurate diagnosis of AD Lack of BBMT-specific clinical utility data likely to be a reason for coverage denial Building a chain of clinical utility evidence to BBMTs could lead to coverage
Diagnosing AD in asymptomatic patients based on abnormal biomarker test results is next frontier for use of BBMTs in clinical settings	Patient demand for testing likely to increase, given recent efforts to educate the public about underdiagnosis of MCI Concerns that a biomarker-based diagnosis of AD in asymptomatic patients will lead to overdiagnosis Professional organizations likely to differ in timing of recommendations for/against use of biomarking testing in asymptomatic persons	Likely to restrict coverage for testing to symptomatic patients Will emphasize that USPSTF recommends against screening for dementia in asymptomatic patients CMS does not cover tests in asymptomatic persons as they are considered screening tests (which are not covered by Medicare unless by specific exemptions)
Economic impact of testing	Budget impact depends on test price and need for confirmatory testing with amyloid PET or CSF biomarkers Economic models could be developed to describe the impact of BBMTs on various clinical test adoption pathways Depends on actionability of test results, leading to choice of interventions that may delay adverse health impacts of advancing AD while also leading to higher treatment costs (e.g., DMTs) Many PCPs reluctant to order tests that will lead to expensive OOP costs for their patients so coverage pathway must be clear	Goal of preserving patient independence and minimizing direct healthcare costs is most feasible in patients diagnosed with early AD Private payers may be less influenced by economic data given member turn-over Economic benefits of delaying complications of AD most likely will accrue to Medicare Intervention costs (e.g., DMTs) likely to be substantial for Medicare if more patients have objective evidence of amyloid burden (although CMS does not consider economic impact of coverage determinations)

Source/Notes: Authors’ analysis of interview data and published literature.

^a^ Not an exhaustive list; potential implications based on published information and interviews. Clinical implications includes both PCPs and specialists.

^b^ Payer implications include both private payers and CMS.

AD: Alzheimer's disease; BBMT: blood-based biomarker test; CDx: companion diagnostic; CMS: Centers for Medicare and Medicaid Services; CSF: cerebrospinal fluid; DMT: disease-modifying therapy; FDA: Food and Drug Administration; LP: lumbar puncture; MCI: mild cognitive impairment; MolDx: Molecular Diagnostics Services Program; NPV: negative predictive value; OOP: out of pocket; PA: prior authorization; PCP: primary care provider; PET: positron emission tomography; USPSTF: United States Preventive Services Task Force.

**Table 2.** Stakeholder ratings of payer considerations.

**Payer considerations**	**Importance**	**Feasibility**
Evidence of Clinical Validity for diverse populations & indications	High	High
Evidence of Clinical Utility for diverse populations & indications	Moderate	Moderate
Limiting testing to specialists	Low	Moderate
Limiting testing to patients being considered for DMTs	Low	High
Require a Coverage with Evidence Development study	Low	Low
Model economic impact (e.g., cost-effectiveness) of BBMTs as a diagnostic test compared to CSF testing or as a triage test	Moderate	High

### Evidence required for use in clinical practice and insurance coverage

*Establishment of clinical validity.* All interviewees stressed that for clinicians to be confident in the use of BBMTs for the diagnosis of AD, there will need to be evidence that BBMTs have been validated in older (e.g., 55 years of age and above) patient groups. Specifically, there needs to be published concordance studies comparing BBMTs with CSF tests that demonstrate that BBMTs have the same or better sensitivity and specificity as CSF testing in diagnosing AD. Additionally, interviewees underscored the need for more information about the clinical validity of BBMTs in patient groups such as the underserved, racial and ethnic minority communities and those with comorbidities and polypharmacy used to manage chronic conditions. Interviewees noted that such clinical validity studies are quite feasible and that several studies are currently underway or recently completed.

*Establishment of clinical utility.* All stakeholders agreed that evidence of clinical utility is essential both for payer coverage and wider clinical use. When discussing this reality, the majority of stakeholders suggested that this could be done by linking the performance of BBMTs to tests that already have been demonstrated to have clinical utility. The consensus amongst most interviewees was that the Imaging Dementia–Evidence for Amyloid Scanning (IDEAS) coverage with evidence development (CED) study established the clinical utility of biomarker testing by demonstrating the impact of the use of amyloid PET on outcomes such as diagnostic change, diagnostic confidence and patient management. Thus, payers might establish a “chain of evidence” that states that ***if*** 1) BBMTs have comparable performance characteristics to CSF testing, and 2) CSF test results are concordant with amyloid PET scans, and 3) the clinical utility of amyloid PET scans has been proven, ***then*** BBMTs have clinical utility. Implications for the diagnostic use case in symptomatic patients is that payers do not need to wait for separate studies documenting the evidence of clinical utility of BBMTs for coverage purposes, which may potentially allow less restrictive coverage policies and thus wider access in clinical care for many patients. An additional consideration for coverage is whether the approval of lecanemab is sufficient to establish the clinical utility of biomarker testing, as positive test results were required to receive treatment, and the current drug labeling does not specify the method for demonstrating amyloid pathology prior to prescribing. However, the lecanemab clinical trials specifically relied on amyloid PET scans and CSF testing and not BBMTs; thus, this conclusion would be based on accepting the chain of evidence argument.

The evidence of clinical utility for BBMTs is less certain for underserved patients and patients with early symptoms of cognitive impairment, who were not adequately represented in the main IDEAS study. Thus, some interviewees suggested that payers may wish to craft policies that encourage clinicians to continue to enroll patients in the ongoing NEW IDEAS CED study that focuses on the association between amyloid PET and patient outcomes.^35^ However, other interviewees thought that another CED study would be burdensome for patients and clinicians, and that the results of the main IDEAS study are likely to be generalizable to all AD biomarker testing (see chain of evidence argument above).

Additionally, while studies have largely established the clinical utility for using BBMTs for the diagnosis of AD in some populations, current evidence is lacking for using BBMTs as a screening test and as a test for monitoring response to DMTs over time. Therefore, interviewees agreed payers would be justified in requiring additional evidence supporting the clinical utility of testing in these applications before covering them.

### Use of BBMTs for DMT eligibility

A key topic covered in the interviews is that payers may wish to restrict BBMT testing to patients undergoing evaluation for beta-amyloid-targeted monoclonal antibody therapies (e.g., lecanemab). However, interviewees pointed out that this approach (while feasible) is not clinically rational nor representative of typical clinical practice. BBMTs would be ordered to make the diagnosis first, then treatment options would be discussed with the patient and family where DMTs would only be one of the alternatives discussed. Moreover, there may be substantial benefit in making the diagnosis of AD irrespective of the possibility of prescribing a DMT. Most stakeholders agreed that patients and caregivers would benefit from knowing their diagnosis and making more informed long-term planning decisions even when provided with the information at a more advanced stage of the disease.

### Potential effects of limiting BBM testing to specialists

Stakeholders agreed that payers might adopt utilization management tools that limit use of BBMTs to memory specialists to minimize the potential for misuse of test ordering and misinterpretation of test results. However, stakeholders noted that the wait times to see a neurologist can extend to more than a year, particularly in underserved areas. Thus, today most patients with memory issues are evaluated in a primary care setting. Primary care providers (PCPs) are accustomed to having to learn about new tests and stakeholders agreed that BBMTs should not be viewed as an exception.

Interviewees agreed that establishing a diagnosis of AD early in the disease course is particularly important given the emergence of DMTs for the treatment of early AD, and thus testing needs to occur at the appropriate time not to miss the DMT treatment window. However, stakeholders also emphasized the need for reframing the impression amongst some PCPs that a more conclusive diagnosis of dementia symptoms in typical patients is not necessary since it would not change their treatment recommendations. Going forward, stakeholders felt that patients would benefit if their PCP received unbiased information regarding the preferred care pathways (e.g., need for referrals, additional testing) for patients with an abnormal or inconclusive BBMT result. The longer-term goal for most stakeholders was to ensure that both PCPs and memory specialists understood that patients and caregivers could benefit from more accurate AD diagnoses, regardless of the stage of the disease. As described by many interviewees, there is clinical and personal utility for making an accurate diagnosis of AD irrespective of eligibility/interest for DMTs; as one interviewee pointed out, “People have a right to know their diagnosis.” Patients and caregivers would benefit from knowing the diagnosis and making more informed long-term planning decisions, assuming the utility of BBMTs is demonstrated.

Most stakeholders recognized that demonstrating these benefits would take many years and therefore acknowledged that developing models that simulated both the costs and outcomes of use of BBMTs in clinical practice could be useful and were definitely feasible. However, they also mentioned that payers often state that they only focus on the clinical evidence when evaluating new tests for coverage determinations, therefore the relative importance of developing economic models could be limited.

### Potential effects of BBMT coverage policies on equitable access to AD diagnosis and clinical management, including new drug treatments

Interviewees noted several ways that coverage policies for BBMTs could mitigate equitable access to AD diagnosis and interventions if the tests are shown to be clinically useful. First, limiting coverage to BBMTs with published evidence of analytic and clinical validity that meet published thresholds (Sensitivity/Specificity/Positive Predictive Value/Negative Predictive Value) for clinical use will help ensure more informative test results for all patients. Adopting different thresholds for a triage test versus a diagnostic test is a practical strategy for balancing patient access to new tests with concerns about inappropriate use. Next, limiting testing to symptomatic patients until there is stronger evidence of the clinical utility of the screening use case benefits all patients by requiring a greater understanding of the sociomedical impact of a biomarker-based definition of AD in asymptomatic persons.

Interviewees also noted that another important avenue that may help broaden access to BBMTs is through coverage for DMTs. Expanding criteria for demonstration of amyloid pathology as part of eligibility assessment for DMTs to include BBMTs (in addition to CSF testing and amyloid PET) would ensure that BBMTs are similarly available to privately insured individuals and Medicare beneficiaries as the other currently covered biomarker testing for AD. Finally, clear coverage policies will avoid replicating coverage confusion that has occurred with other AD biomarker testing by defining a clear evidence pathway—proactively clarifying the coverage determination process would benefit all patients.

Conversely, coverage policies may also exacerbate inequities in AD diagnosis and clinical management. The suggestion that payers might limit AD BBMT testing to specialists generated the strongest negative reactions from all interviewees, given that PCPs currently diagnose most cases of AD, they are familiar with adopting new lab tests, and an insufficient number of specialists to meet the need for AD testing and diagnosis.

A lack of broad coverage for BBMTs means that the window of opportunity for treatment closes for many patients as they progress from mild to moderate AD before, they are even diagnosed, making them ineligible for current DMTs. This is likely to be more common in underserved patients who are more likely to have delays in diagnosis. While probably feasible as a utilization management tool to ensure interpretative accuracy and specialty follow-up care, PCPs currently diagnose most cases of AD and are likely to continue to do so, given the limited supply of specialists and their current geographic distribution. Thus, limiting ordering of BBMTs to neurologists or dementia specialists could potentially widen existing disparities in care.

Lastly, if coverage for BBMT testing is limited to patients who are being evaluated for potential DMT therapy (and therefore only have mild cognitive symptoms), this would disproportionately impact underserved patients who typically do not have access to DMTs as easily as other patients. As described by many interviewees, there is clinical and personal utility for making an accurate diagnosis of AD irrespective of eligibility/interest for DMTs as “People have a right to know their diagnosis.” Patients and caregivers would benefit from knowing the diagnosis and making more informed long-term planning decisions, assuming the utility of BBMTs is demonstrated.

## Discussion

We assessed the importance and feasibility of a broad range of typical payer coverage considerations in terms of their likely impact on the clinical management of diverse patient groups with symptoms of cognitive impairment. As with other biomarker tests, payers will require evidence of clinical validity and clinical utility of BBMTs as part of their coverage determination processes.^36,37^ These payer evidence requirements are not unique to BBMTs, but given the recent availability of DMTs, the current climate of AD underdiagnosis and misdiagnosis, and the potential for improving the accuracy and timeliness of AD diagnosis in primary care settings, there is an urgent need to proactively address coverage considerations in a manner that does not exacerbate current health inequities.

Lessons about the introduction of new tests can be learned from the introduction of cancer precision medicine tests (e.g., tumor profiling tests), which demonstrate not only the importance of payer coverage decisions, but also the need for physician and patient education. Studies demonstrate that minority groups are significantly less likely to have tumor profiling tests ordered^38^ to help manage their cancers, even in the Medicare population^39^ where the tests are covered for advanced disease. Regarding the diagnosis and treatment of AD, stakeholders emphasized the need for closing knowledge gaps particularly given the widespread perception in primary care that there are no effective interventions for AD.

There is significant variability in the performance characteristics of currently marketed tests.^22^ The indication for testing is a critical factor in determining whether BBMTs have sufficient evidence of their sensitivity, specificity, and positive and negative predictive values to establish clinical validity. Our interviewees confirmed the need for additional clinical validity studies in the intended use populations, specifically in diverse groups with respect to race, ethnicity, age, and comorbidities. Currently, there are residual evidence gaps for the interpretation of BBMTs in these communities that will need to be addressed. The specific indication for testing (diagnostic test, triage test, or screening test) will affect these evaluations and acceptable thresholds for classifying tests as positive or negative. Specifically in the screening context, the US Preventive Services Task Force (USPSTF) will play a major role in reviewing the evidence of the clinical utility of BBMTs, so the Task Force should ensure that there is sufficient expertise on the panel in the management of neurological diseases such as AD. If USPTF determines that the evidence of BBMTs merits an “A” or “B” rating, then payers are obligated to cover the test when used for screening. However, this process will likely take years and so is not likely to positively affect payer coverage determinations in the near future.

Currently, there is robust published evidence of clinical validity for both diagnostic and triage use cases in predominantly European ancestry populations, while there is limited evidence to support use of BBMTs as screening tests. Furthermore, CMS does not currently cover screening tests except by legislative exception, and the USPSTF currently concludes that there is insufficient evidence to determine whether the benefits of screening for cognitive impairment in older adults outweigh the harms.^40^ While CMS does not consider the economic impact of a new test when making a coverage determination, many private payers need to consider how tests that may lead to substantial downstream resource utilization will affect per-member per-month costs to the plan. Therefore, economic models of the relative costs and benefits of BBMTs would also assist some payers in their coverage considerations.^41^

The CED study that was considered by the majority of stakeholders to have established the clinical utility of amyloid PET scans in the diagnosis of AD could be generalizable to all biomarker testing assuming adequate concordance across various test modalities. While most interviewees concurred with this position, a few stakeholders wanted to see additional clinical utility studies focused on BBMTs, conducted in primary care settings.

Given the shortage of memory specialists, primary care physicians are very likely to be the principal users of BBMTs and limiting testing coverage authorization to specialists could be harmful to patients, particularly the underserved. Reliance on PCPs will require additional training, more time to spend with patients and implementation of multidisciplinary care models.^42^ For example, if PCPs do not have adequate knowledge about the current evidence base for BBMTs, there is the possibility that the tests will be implemented for use cases where the evidence is not sufficient to establish clinical validity (e.g., use as a screening test for asymptomatic patients).

Even if PCPs have adequate training to order BBMTs appropriately, the system will require additional capacity to perform confirmatory testing, particularly if BBMTs are being used as a triage test to stratify patients for secondary care by specialists. An additional issue that will require further study is how payers will address coverage for reflex testing if the initial BBMT test result is not clearly positive or negative. For example, questions such as under what circumstances would they pay for a second BBMT test or pay for a CSF test and over what time period will need to be tackled. Finally, while today, there is insufficient evidence to use BBMTs as monitoring tests, patients treated with DMTs want to know if they are responding, and BBMTs are being studied for this indication.^43^

Another pressing issue highlighted by interviewees is that clinicians do not routinely order biomarker testing to make the diagnosis of AD, particularly in underserved populations. Thus, stakeholders stressed the need for education from unbiased sources about how to use BBMTs, how to interpret the results, and when to refer to specialty care. Additional studies are needed to assess best practices for implementing BBMTs in primary care, including tracking appropriate referrals to specialists and memory clinics, patient willingness to undergo testing, and satisfaction with diagnostic procedures.

Finally, stakeholders were concerned that coverage barriers would cause delays in care that would put some early-stage dementia patients beyond the window for treatment with disease-modifying therapies as they progress from mild to moderate AD. This is one of the most emphasized issues by interviewees as summarized by the opinion of one expert, “Limiting access to neurologists or dementia specialists might do wonders for interpretative accuracy and follow-up benefit, but also be very detrimental to racial and SES equity.”

Our study has several limitations, including the reliance on a relatively small number of expert interviews that may not fully reflect relevant perspectives, but which provide valuable information given the lack of prior studies. There was saturation for many of the key coverage considerations discussed, particularly agreement about the gatekeeper role of PCPs and the implications for patient access if coverage was limited to specialists. However, not all stakeholders agreed with the biomarker-based definition of AD advanced in the recent update to the Alzheimer's Association guidelines for diagnosis and staging of AD. These clinicians emphasized the importance of continuing to rely on a careful clinical evaluation of cognitive functioning and not labeling individuals as having AD at an asymptomatic phase. Finally, considerations of billing and coding for BBMTs was considered out of scope and not discussed in detail with stakeholders.

### Conclusions

We are at an inflection point given the anticipated approval of more DMTs in the near future, as well as growing public interest in diagnosing AD early when there is the potential for getting the most benefits from DMTs or other interventions.^44,45^ Payer coverage will affect patient access to biomarker testing for AD and be central to implementing emerging diagnostic guidelines. Given existing inequities in AD diagnosis and treatment, it would be beneficial to anticipate and plan for the many important policy issues triggered by BBMTs, such as considerations for coverage and reimbursement.^46^ Early consideration of coverage issues can help address the potential effects of payer decisions on equitable patient access.

## Supplemental Material

sj-docx-1-alz-10.1177_13872877251329394 - Supplemental material for Alzheimer's disease blood-based biomarker testing: A stakeholder-informed assessment of coverage considerations Supplemental material, sj-docx-1-alz-10.1177_13872877251329394 for Alzheimer's disease blood-based biomarker testing: A stakeholder-informed assessment of coverage considerations by Patricia A Deverka, Jalayne J Arias, Grace A Lin, Jessica Zwerling and Kathryn A Phillips in Journal of Alzheimer's Disease
