# Supplementary material for: Alzheimer's disease blood-based biomarker testing: A stakeholder-informed assessment of coverage considerations
Source: J Alzheimers Dis. 2025 Apr 1;105(2):433–42. doi: 10.1177/13872877251329394 (PMC12143641; doi:10.1177/13872877251329394)
Supplement: sj-docx-1-alz-10.1177_13872877251329394 - Supplemental material for Alzheimer's disease blood-based biomarker testing: A stakeholder-informed assessment of coverage considerations [file sj-docx-1-alz-10.1177_13872877251329394.docx]

**Supplemental Material**

**Alzheimer’s disease blood-based biomarker testing: A stakeholder-informed assessment of coverage considerations**

**Methods**

*Literature search*

Investigators conducted a search for eligible sources in PubMed, using the following search terms: ((“alzheimer disease”[MeSH Terms] OR (“alzheimer”[All Fields] AND “disease”[All Fields]) OR “alzheimer disease”[All Fields] OR “alzheimer’s”[All Fields]) AND ((blood-based[All Fields] AND (“biomarkers”[MeSH Terms] OR “biomarkers”[All Fields])) OR ((“plasma”[MeSH Terms] OR “plasma”[All Fields]) AND (“biomarkers”[MeSH Terms] OR “biomarkers”[All Fields]))) AND clinical[All Fields]) AND and systematic review[All Fields].

We excluded studies of cerebrospinal fluid biomarkers alone, blood-based biomarkers other than amyloid and tau (e.g., neurofilament light and glial fibrillary acidic protein), and neurodegenerative diseases other than Alzheimer’s disease. We considered sources published in English in the past 5 years (2018-23), supplemented by specific recommendations for articles recommended by co-authors based on their expertise as well as the pilot interviewee.

Stakeholders (n = 12)

| **Perspective** | **Number** |
| --- | --- |
| Neurologist | 5 |
| Primary Care Physician | 2 |
| Geriatrician | 2 |
| Health equity expert | 2 |
| Clinical researcher | 9 |
| Patient advocate | 1 |
| Test developer/laboratory medicine expert | 3 |
| Former payer | 1 |
| TOTAL | Exceeds 12 because interviewees typically represented multiple perspectives |

**Interview Guide**

**Stakeholder perspectives on key considerations for payer coverage of blood-based biomarker tests for diagnosing patients with Alzheimer’s Disease**

*NIH-funded project*

*Interviews are confidential and only attributable to individuals upon request in the final report*

The purpose of these interviews with Alzheimer’s Disease (AD) experts (clinicians, researchers, patient advocates, test developers) and payers is to inform the development of key considerations for obtaining payer coverage, since this a critical step on the journey to clinical adoption of blood-based biomarkers (BBMs). In order to create a more predictable coverage pathway, we are initially focusing on diagnostic indications for BBMs, but will also discuss use as a triage test to identify patients for amyloid PET scans or CSF testing and as a screening test. Our research team has extensive experience with the criteria used by payers when making coverage determinations for novel precision medicine tests, but we predict that BBMs may require unique considerations that must be informed by knowledge of both the science of BBM’s and clinical management of patients with AD. We plan to explore these issues with the questions below.

**Interview Questions**

1. When you think about insurance coverage for BBMs, what are the challenges that you foresee?
   1. Are there particular aspects of BBM that would make coverage different from PET scans or CSF testing?
   2. What kind of evidence still needs to be generated for BBMs to be covered and used in routine care?
   3. Are there implementation challenges that you see for BBMs? (e.g., availability, quality control, results interpretation, pre-/post-test counseling challenges?)
   4. Are there additional challenges for disadvantaged populations?
2. What are your reactions to the following list of key considerations for payers when evaluating BBMs for potential coverage? Probes:
   1. Conduct additional Clinical validity and Clinical Utility studies in diverse populations
      1. New IDEAS (Imaging Dementia – Evidence for Amyloid Scanning) study opportunities?
      2. Are clinical practice guidelines (such as the recent NIA-AA guidelines) an appropriate type of evidence for payers to rely on where evidence may be emerging or changing rapidly?
         1. If yes, how should guidelines be updated in a timely manner to affect payer coverage?
   2. Model cost-effectiveness of BBMs vs CSF testing for both diagnosis and/or for monitoring?
   3. Engage patients/caregivers in research study planning?
   4. Limit AD testing/diagnosis to dementia specialists (neurologists and geriatricians) in collaboration with PCPs?
      1. Would you feel comfortable with the PCP ordering BBMs and administering cognitive tests? Why or why not?
      2. Implications for prior authorization by limiting BBMs to neurologists?
   5. Consider a CED (coverage with evidence development) study?
   6. Limit testing to patients being considered for amyloid blocker drugs?
3. Partner with laboratories and/or diagnostics companies to develop preferred lab networks to ensure test quality and patient safety? [Dropped due to interviewee insufficient knowledge of topic]
4. Please rate the relative importance and feasibility of each consideration we just discussed (Likert scale 1-5 for each construct)
   1. 1=Very important; 2 = Important; 3 = Moderately important; 4 = Slightly important; 5= Not important [1-2 = High; 3 = Moderate’ 4-5 = Low]
   2. 1 = Definitely feasible; 2 = Very probably feasible; 3 = Probably feasible; 4 = Possibly feasible; 5 = Not feasible [1-2 = High; 3 = Moderate’ 4-5 = Low]
5. How might payer coverage policies affect access to AD diagnosis and treatments? Affect equity considerations?

| **Consideration** | **Importance** | **Feasibility** |
| --- | --- | --- |
| CV studies |  |  |
| CU studies |  |  |
| CE model |  |  |
| Engage pts/caregivers in research |  |  |
| Limit AD testing to specialists |  |  |
| CED study |  |  |
| Limit BBM testing to eval for amyloid blockers |  |  |
